# The Influence of Different Surface Cleansing Agents on Shear Bond Strength of Contaminated Lithium Disilicate Ceramic: An In Vitro Study

**DOI:** 10.1155/2021/7112400

**Published:** 2021-08-10

**Authors:** Taksid Charasseangpaisarn, Pattarawadee Krassanairawiwong, Chanidapa Sangkanchanavanich, Atima Kurjirattikan, Kanyarak Kunyawatyuwapong, Natlada Tantivasin

**Affiliations:** College of Dental Medicine, Rangsit University, Pathum Thani 12000, Thailand

## Abstract

**Materials and Methods:**

Seventy LDS specimens were randomly divided into seven groups. The first group was noncontaminated surface (PC). The six other groups were contaminated with the saliva and silicone disclosing medium and treated with no surface cleansing agent (NC); phosphoric acid (PO); Ivoclean (IV); sodium hydroxide solution (NA); Restorative Cleansing Agent (RC); and hydrofluoric acid (HF). Then, LDS specimens were cementated with Panavia V5 to resin composite rod. Each specimen was subjected to an SBS test. The modes of failure was inspected under light microscope. The surface element of each group was examined by SEM-EDS.

**Results:**

The results were analyzed with one-way ANOVA and Tamhane's T2. The mean SBS value of NC was significantly lower than others (*p* < 0.05), and HF was significantly higher than others (*p* < 0.05). However, PC, PO, IV, NA, and RC were not significantly different from each other (*p* > 0.05). The mode of failure was mostly adhesive failure in every group. The surface showed similar amount of elements in every group.

**Conclusions:**

The SBS of LDS was reduced by saliva and silicone disclosing medium contamination which can be restored using acid- and alkaline-based surface cleansing agents before the cementation procedure.

## 1. Introduction

Nowadays, esthetics is one of the factors in material selection for dental restorations. Recently, there has been a significant growth in using lithium disilicate (LDS) in dental practices. LDS is well known for its mechanical properties and its versatility in dental uses. It can be used to fabricate various types of fixed restorations, including single crowns and multiple-unit bridges [1]. Furthermore, the advantage of LDS over other materials is that it has extremely low fracture rates. It can withstand a ration fatigue of 1 million cycles at loads of 1,000 N [2]. The study showed that LDS Press had higher fatigue failure load and number of cycles for failure than LDS-CAD when the Monobond Etch and Prime system was used. However, when conditioned with hydrofluoric acid and silane, the difference in failure is not significant [3].

In common dental practices, fixed restorations are clinically tried in the patient's mouth prior to cementation. In this step, the restoration is inevitably contaminated with both the saliva and silicone disclosing medium. Saliva is a slightly acidic mucoserous exocrine secretion in the oral cavity. It is composed of various electrolytes, for example, sodium, potassium, calcium, magnesium, bicarbonate, and phosphates. Saliva also consists of mucins, nitrogenous products, and macromolecule proteins such as immunoglobulins, proteins, and enzymes [4]. The silicone disclosing medium, also known as silicone fit indicator, is a material used to detect the interference on the intaglio of a crown. This helps increase the fit of the crown to the prepared abutment by indicating the spots of interference, which can then be corrected by the operator. After repeated use of the silicone fit indicator and correction of the interferences, the dentists can achieve a complete seating of the crown in the patient's mouth [5, 6]. Unfortunately, these contaminations significantly decrease the bond strength between cements and the adhesive surface of the restoration and may result in reduced restoration's longevity [7, 8].

Saliva protein deposits on the restoration surface and forms acquired pellicles which change the wettability and surface free energy of the crown. Thus, degradation of adhesive bonding occurs [8–10]. The silicone disclosing medium leaves some residuals on the inner surface of the restorations which lead to a decrease in its bond strength with cements, both micromechanically and chemically. The porosities created on the adhesive surface of the restorations by surface treatment are occupied by the silicone, preventing micromechanical interlocking between ceramics and cements. Moreover, its by-products from Si–OH groups can also impair the free surface energy of the porcelain [11]. Therefore, in order to overcome potential clinical problems, the ceramic surfaces should be cleaned from any contaminants prior to the cementation process [12]. According to the previous studies, cleansing the contaminated LDS crown results in reliable improvement of bond strength. Chemical surface cleansing can be divided roughly into acid- and alkaline-based group. Acid-based group includes hydrofluoric acid (HF) and phosphoric acid (H_3_PO_4_), while alkaline-based group is consisted of Ivoclean and sodium hydroxide solution. The main mechanism of cleansing agents is using either their alkalinity or acidity to remove residual organic contaminants and result in successful bond strength recovery between ceramic and adhesive [13–15]. Hydrofluoric acid further decontaminates the surface by alteration of the surface microstructure and simultaneously increases the surface roughness [16]. Thus, the purpose of this study was to investigate the influence of various cleansing agents on the SBS value of contaminated LDS surface.

The hypothesis of this study was that different cleansing agents could recover the SBS of LDS restorations which have been contaminated with the saliva and silicone disclosing medium.

## 2. Materials and Methods

### 2.1. Specimen Preparation

LDS-based ceramic blocks (IPS e.max CAD MT A1, Ivoclar Vivadent, Schaan, Lichtenstein) were cut with low-speed cutting machine (ISOMET 4000, Beuhler Ltd, Illinois, USA) into seventy equal square-shaped specimens 7.25 × 7.25 × 2 mm^3^ in size. Then, the LDS specimens were sintered in the furnace (Programat P300; Ivoclar Vivadent) at 840°C for 10 minutes. After that, the specimens were placed in the polyvinyl chloride (PVC) tubes and bonded with self-cured acrylic resin (Unifast Trad, GC Corporation, Tokyo, Japan). The embedded ceramic specimens were polished with 600-grid metallographic abrasives using a Nano 2000 polisher (Pace Technologies, Arizona, USA) under hydraulic pressure for 1 minute and then immersed in an ultrasonic bath for 15 minutes.

**Figure 1:**
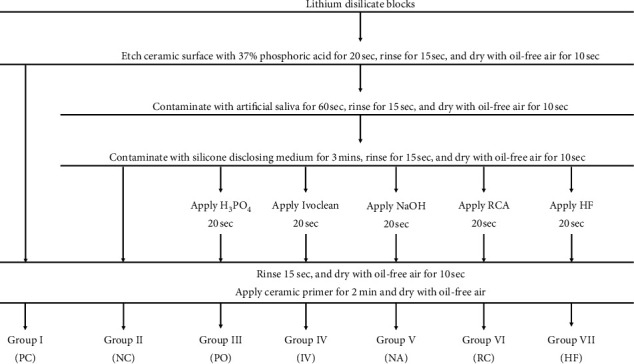
The protocols for surface treatment of each group.

In every group of the specimens, the ceramic surface was cleaned with 37% phosphoric acid (Scotchbond Etchant, 3M ESPE, Minnesota, USA) using a microbrush (Dentsply, New York, USA) for 20 seconds. Then, the ceramic was rinsed with water for 15 seconds and dried with oil-free air for 10 seconds. After that, the specimens were divided randomly into 7 groups, Figure 1.

### 2.2. Surface Contamination

In group 1, the specimens were left noncontaminated as the positive control group (PC). In group 2–7, the specimen surfaces were applied with artificial saliva (Faculty of Dentistry, Chulalongkorn University, Bangkok, Thailand) for 60 seconds, rinsed with water for 15 seconds, and dried with oil-free air for 10 seconds. The specimens were then contaminated with a Fit Checker (GC Corporation, Tokyo, Japan) for 3 minutes. After that, the silicone was removed and the specimen surfaces were rinsed with water for 15 seconds. Then, they were dried with an oil-free air for 10 seconds. In group 2, the specimens were not clean as the negative control group (NC). In group 3–7, the specimens were cleaned with different cleansing agents as follows (Figure 1):

Group 3, phosphoric acid (PO): the specimens were cleaned with 37% phosphoric acid (Scotchbond Etchant) for 20 seconds, rinsed with water for 15 seconds, and air dried for 10 seconds with oil-free air.

Group 4, Ivoclean (IV): the specimens were cleaned with universal cleansing paste (Ivoclean^TM^; Ivoclar Vivadent, Schaan, Liechtenstein) for 20 seconds using a microbrush, then rinsed with water for 15 seconds, and dried for 10 seconds with oil-free air.

Group 5, NaOH solution (NA): the specimens were immersed in a container with 0.5 M NaOH solution for 20 seconds, rinsed with water for 15 seconds, and air dried for 10 seconds with oil-free air.

Group 6, Restorative Cleansing Agent (RC): the specimens were cleaned by applying a restorative cleansing agent (College of Dental Medicine, Rangsit University, Pathum Thani, Thailand) for 20 seconds, rinsed with water for 15 seconds, and air dried for 10 seconds with oil-free air.

Group 7, hydrofluoric acid (HF): the specimens were cleaned with 5% HF IPS ceramic etching gel (Ivoclar Vivadent, Schaan, Liechtenstein) for 20 seconds, rinsed with water for 15 seconds, and air dried for 10 seconds with oil-free air.

The composition of the surface cleansing agents is described in Table 1.

### 2.3. Bonding the Specimens

The seventy cylindrical resin composite specimens (Filtek Z350 XT A3, 3M ESPE, Minnesota, USA) size 3 mm in diameter were fabricated and randomly divided into seven groups. The LDS specimens were treated with Clearfil Ceramic Primer (Kuraray Noritake, Tokyo, Japan) and gently dried with oil-free air. Then, the resin composite specimens were bonded to LDS with Panavia V5 (Kuraray Noritake, Tokyo, Japan) under 1 kg load using a Durometer (ASTM D 2240 TYPE A, D PTC Instrument, USA). The excess cement was removed. The specimens were light-cured with an LED light cure unit (DEMI™ Plus, Kerr Dental, California, USA) for 40 seconds. The load was applied on the specimen for 8 minutes to ensure complete setting of cement.

### 2.4. Shear Bond Strength (SBS) Test

**Figure 2:**
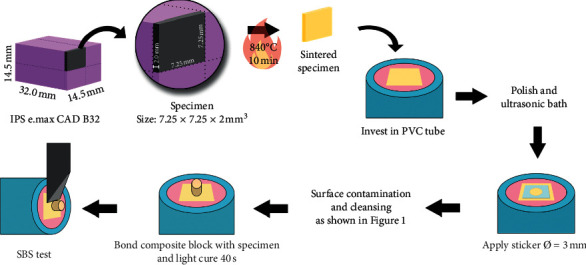
The illustration of the overall procedures.

The specimens were immersed in 37^o^C water in an incubator for 24 ± 2 hours prior to the SBS test. The SBS test was performed with a universal testing machine (EZ-S, Shimadzu, Kyoto, Japan) with a crosshead speed of 0.5 mm/min. The shear force was applied to the ceramic-resin composite interface until fracture occurred, and the surfaces were examined. The data were analyzed by one-way ANOVA and Tamhane's T2 using SPSS 16.0 for Mac OS (SPSS Inc., Illinois, USA). The overall procedures are illustrated in Figure 2.

### 2.5. Evaluation of Modes of Failure

All debonded specimens were analyzed with a light microscope (SZ-61, Olympus, Tokyo, Japan) using 25X magnification to grossly categorize the modes of failures into 3 types as follows:

1. Adhesive: the failure occurred at the interface between two different surfaces
2. Cohesive: the failure occurred within the material
3. Mixed failures: the failure occurred in both adhesive and cohesive with at least 25% of either type [17]

Then, the specimens that could not be categorized into any of the mentioned groups underwent fractographic examination by using a scanning electron microscope (JSM-5410LV; JEOL, Tokyo, Japan) with 70X magnification.

### 2.6. Scanning Electron Microscopy (SEM) and Energy-Dispersive Spectroscopy (EDS) Analysis

A specimen from each group was prepared following the method up to the surface cleansing procedure for scanning electron microscopy (SEM) analysis. The SEM was used for analysis of the morphology of the LDS surfaces to observe the surface characteristic, and EDS was also performed to determine the ratio of the element on the surface of each specimen.

## 3. Results

**Figure 3:**
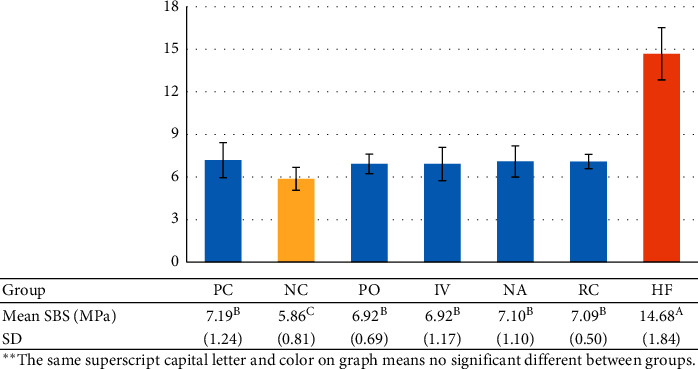
The mean SBS and SD of each group.

The data were analyzed using the Shapiro–Wilk test, and normal distribution was shown in all groups. Thus, one-way ANOVA and Tamhane's T2 tests were used for the comparison test. The mean SBS values of all groups were summarized as shown in Figure 3. One-way ANOVA showed significant differences between groups. From Tamhane's T2, the mean SBS values of every test group were shown to be significantly higher than NC (*p* < 0.05). Every group treated with surface cleaning agents showed slightly lower SBS values than PC with no significant difference (*p* > 0.05), exception of HF which showed the highest SBS values among all groups (*p* < 0.05).

**Figure 4:**
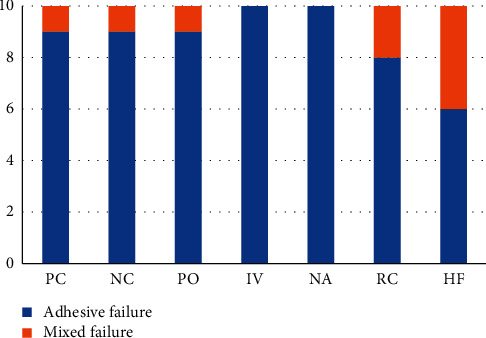
The number of specimens classified by the mode of failure in each group.

After evaluating the specimens under the stereomicroscope, the specimens in all groups were mostly found to undergo adhesive failure between the LDS specimen and resin cement, as shown in Figure 4. The number of adhesive failures was the least in HF with the highest mixed failure.

**Figure 5:**
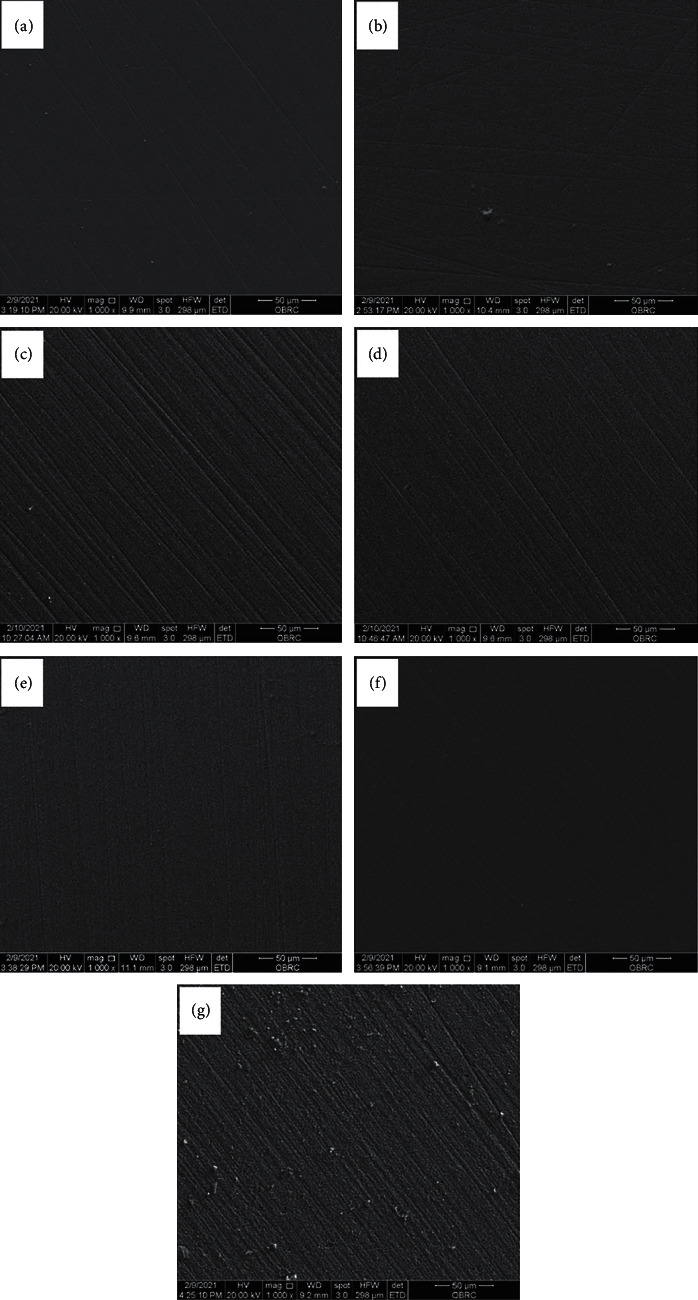
Surface characterization without cementation of PC (a); NC (b); PO (c); IV (d); NA (e); RC (f); and HF (g).

The surface characteristics of contaminated LDS after application of different surface cleansing agents investigated with SEM are shown in Figure 5.

## 4. Discussion

LDS is composed of 70% crystalline content with a needle-like structures embedded in a matrix containing mainly SiO_2_, Li_2_O, Al_2_O_3_, K_2_O, P_2_O_5_, and other oxide substitutes. Prior to cementation, surface treatment and silanization are required. The recommended protocol of surface treatment in LDS is the application of 5% hydrofluoric acid etching for 20 seconds [14]. Acid dissolves the glassy phase in ceramic, leading to an increase in surface roughness and, consequently, a micromechanical interlocking between the ceramic and resin cement, whereas silane, a bifunctional molecule, promotes ceramic-resin adhesion and reduces the contact angle and increases the wettability of the ceramic surface. Former studies showed that improvement in bond strength was significant after silane application [18]. However, the bond strength of the restoration can be minimized by various contaminations such as the saliva, blood, and silicone disclosing medium.

From the result of this study, it could be seen that NC showed statistically lower mean SBS value compared to PC. This implied that the contamination from both the saliva and silicone disclosing medium reduced the bond strength between the surface of LDS restoration and resin composite bonded with resin cement (Panavia V5). The result obtained in this study correlated with the previous studies [7, 8, 15]. The surface of LDS could be clinically contaminated with the saliva and silicone disclosing medium during the try-in procedure. Saliva is composed of various electrolytes such as sodium, potassium, calcium, and proteins [4]. Saliva may interfere with bond strength by adhering to the surface of LDS which decreases wettability and surface free energy of the restoration. The silicone disclosing medium is a modified polyvinyl-siloxane which may not be completely removed with either ultrasonification or acidic etchants due to its poor reactivity. The residues retained in the microporosities may decrease the surface energy and bonding area [5, 6, 19].

The contaminated surfaces of the restorations treated with different cleaning agents (PO, IV, NA, and RC) showed that the recovered SBS was not statistically different from the noncontaminated surface (PC). This indicated that surface cleansing agents might be able to effectively clean the LDS surface contaminated with the saliva and silicone disclosing medium. The result was supported by previous studies that cleansing the intaglio surface before cementation is recommended to prevent a reduction of bond strength after contamination [8–10]. However, different surface cleansing agents had different impacts on bond strength recovery.

Two mechanisms of action of widely used surface cleansing agents are alkaline based and acid based. Alkaline-based agents used in this study included Ivoclean, NaOH solution, and Restorative Cleansing Agent. The acid-based agents used were phosphoric acid and hydrofluoric acid, as shown in Table 1. In alkaline-based groups, the main mechanism is from their alkalinity which removes residual organic contaminants. Ivoclean is a highly alkaline universal cleansing paste containing zirconia particles. It was claimed to have strong affinity towards the phosphate group which results in removal of contaminants in saliva. However, in the previous study on zirconia, the shear bond strength of contaminated zirconia cleaned with 0.5 M NaOH solution for 20 seconds was restored to the level which has no significant difference as compared to the noncontaminated zirconia [13]. From the result of this study, NaOH solution was able to restore bond strength. Thus, alkalinity was the main mechanism of removing the contaminant on contaminated zirconia [14]. This was in agreement with the previous study by Attia and Ebeid, which found Zirclean could effectively clean contaminated translucent zirconia restoration [20]. The main composition of Zirclean is potassium hydroxide (KOH) without zirconia particles, which can also remove organic contaminants due to its alkalinity. NaOH solution is a strong base which could be toxic to tissues. NaOH solution in the liquid form which was difficult to handle due to the restoration must be immersed in the solution [13]. Thus, RCA (restorative cleansing agent) which is mainly composed of sodium hydroxide (NaOH) in the gel form was developed. In this form, less amount of the cleansing agent is needed per use. Moreover, its superior viscosity requires no container for immersion of the restoration and allows better handling than NaOH solution.

**Figure 6:**
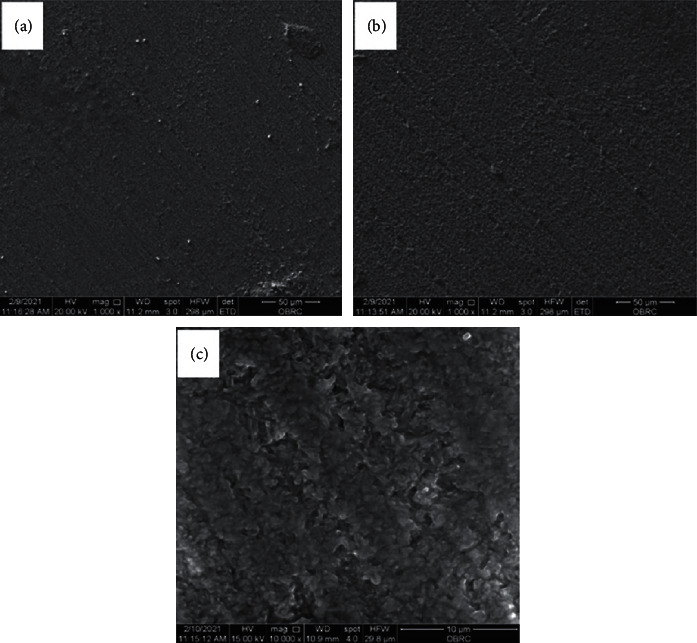
Surface characterization of specimens not etched with hydrofluoric acid 1,000x magnification (a); etched with hydrofluoric acid for 20 seconds at 1,000x magnification (b); and etched with hydrofluoric acid for 20 seconds at 10,000x magnification (c).

Acid-based groups such as phosphoric acid removed residual organic contaminants before the bonding procedure, providing a clean surface which resulted in a successful bonding between ceramics and adhesives [15]. The result is the same with that of alkaline-based cleansing agent. The HF group showed the highest mean SBS value which was statistically significant to every test group. The higher bond strength of the HF group was a result of micromechanical interlocking created by the surface roughness of the ceramic. HF is an acid that can dissolve the glassy matrix containing silica (SiO_2_), silicates (SiO_4_^4-^), and leucite crystals (K_2_O•Al_2_O_3_•4SiO_2_) in silica-based ceramic [16, 21]. HF did not only provide more surface area for resin bonding but also increased the wettability for bonding. Hence, the mean SBS value of the hydrofluoric acid group was higher than that of the other groups. The surface of LDS was observed under an SEM at 1000x magnificent and 10000x magnification to identify the surface topographic change before and after etching with 5% HF for 20 seconds, as shown in Figure 6.

Compared to adhesive failure, mixed failure indicated higher ability of adhesion between resin cement and the two surfaces: the LDS and resin composite. According to the result of this study, the group with the greatest number of mixed failure was HF; however, the value was insignificant. This corresponded to the superior mean SBS value of HF. On the other hands, other groups underwent merely adhesive failure. Adhesive failure indicated that the bond strength between resin cement and other surfaces was not as strong as one within the materials themselves. According to the previous studies, the aging simulation by thermocycling reduced mean bond strength of the LDS specimens. This could be explained by the difference in the coefficient of thermal expansion between the two substrates which induced the stress in adhesive materials [22, 23]. Thus, the effect of aging simulation may be investigated in the future study.

The SEM of all groups was performed at 1000x magnification to compare the surface topography. Energy-Dispersive Spectroscopy (EDS) was also performed at 1000x magnification to determine the elements found on a particular area of the surfaces. From SEM, it was found that the surfaces of PC, NC, PO, IV, NA, and RC were similar in terms of surface character (Figure 5). However, the surface of HF showed higher roughness than the others (Figure 6). Moreover, it could be observed in HF that there were dust-like particles on the surface which was suspected to be fluorosilicate salt dissolved from the glassy phase of LDS [4].

**Figure 7:**
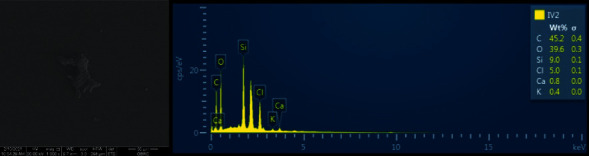
Fit checker residual observed under an SEM and elements detected at the area with a fit checker residual under an EDS.

In some areas of contamination, the silicone disclosing medium residual was also observed. The EDS was performed on the suspected area to identify the contamination. There were certain amounts of Si and C (Figure 7) which were the main compositions of the silicone disclosing medium. This was in agreement with previous studies in which the remnants of the silicone disclosing medium still remained after cleaning the contaminated surface with different cleansing agents. However, these residuals did not have a significant impact on the bond strength of restoration after cleansing protocols were performed [19, 24].

Furthermore, the EDS was used for elemental investigation of the surfaces in all groups. Theoretically, carbon (C) is supposed to be found in the contaminated specimens due to the presence of carbon as parts of protein in the saliva. However, from the result of this study, carbon was found upon EDS investigation in every group, including PC which was not contaminated by the saliva and silicone disclosing medium. According to Kalinkin et al., it was found that the presence of a carbon component in ceramic resulted from the milling process. There was a mechanosorption of CO_2_ molecules from the atmosphere penetrating into the silicate matrix and then dissolving in the matrix forming a carbonate-silicate phase [25]. However, the silicon (Si) did not be the element for investigation of contamination since the main composition of LDS ceramic is also Si. From this study, NC which should theoretically contain higher amounts of organic substances showed similar values of findings with PC. Therefore, EDS may not be the best tool for studying the contamination of LDS fabricated by milling or CAD. An X-ray photoelectron spectrometer (XPS) which detects the elements on a surface in numbers may be a more suitable tool. The limitation of this study was that other materials, such as metal or zirconia ceramic, and other types of bond strength, such as tensile, microtensile, or microshear bond strength, were not included. Hence, further investigation regarding these topics may be beneficial. Another limitation of this study was that the study is an *in vitro* study, and more clinical studies may be needed to extrapolate the findings.

## 5. Conclusions

Prior to cementation, contamination with the saliva and silicone disclosing medium is inevitable to the intaglio of the restoration.

1. Saliva and silicone disclosing medium leave residual contaminants on the LDS surface, causing a significant decrease in shear bond strength
2. The reestablishment of bond strength can be achieved by using surface cleaning agents before the cementation procedure

## Figures and Tables

**Table 1:** The composition and pH of cleansing agents using in the study.

Cleansing agents	pH	Compositions
Scotchbond Etchant	2.7	Orthophosphoric acid, water, poly (vinyl alcohol)
Ivoclean	13.0–13.5	Sodium hydroxide, ZrO_2_, water, polyethylene glycol, pigments
NaOH solution	13.0–13.5	Sodium hydroxide, water
Restorative Cleansing Agent	13.0–13.5	Sodium hydroxide, water, poly (acrylic acid) polymer, glycerine, pigments
IPS Ceramic Etching Gel	2	Hydrofluoric acid

## Data Availability

The data used to support the findings of this study are available from the corresponding author upon request.
